# Evaluation of the safety of short-term follow-up CT for the management of consolidation in lung cancer screening

**DOI:** 10.1007/s00330-025-11609-x

**Published:** 2025-05-02

**Authors:** Emily C. Bartlett, Ley Chan, Justin Garner, Sujal R. Desai, Samuel V. Kemp, Simon Padley, Bhavin Rawal, Carole A. Ridge, James Addis, Anand Devaraj

**Affiliations:** 1 Department of Radiology, Royal Brompton Hospital, London, UK; 2 National Heart and Lung Institute, Imperial College, London, UK; 3 Department of Respiratory Medicine, Royal Brompton Hospital, London, UK; 4 Department of Respiratory Medicine, Nottingham University Hospitals NHS Trust, Nottingham, UK

**Keywords:** Early detection of cancer, Lung, Diagnostic screening programmes, Computed tomography

## Abstract

**Objectives:**

Focal consolidation on CT may be inflammatory or malignant, and PET-CT imaging is rarely discriminatory. Furthermore, consolidation may demonstrate spontaneous resolution obviating the need for PET-CT imaging. This retrospective study sought to assess the safety and cost-effectiveness of short-interval 6-week follow-up CT for consolidation in a lung cancer screening programme.

**Methods:**

Between January 2019 and January 2024, participants in a regional lung cancer screening programme with focal indeterminate consolidation underwent a 6-week repeat CT rather than immediate PET-CT and invasive investigation. The proportion of participants with non-resolving consolidation, the risk of malignancy in consolidation at a 6-week follow-up, and the risk of upstaging over a 6-week delay were determined. Cost savings were estimated from National Health Service reference costs.

**Results:**

In 10,247 CT studies, focal indeterminate consolidation was detected in 113 participants (1.1%) (mean age 68 years, range 55–76, 65 males). Consolidation spontaneously resolved at 6 weeks in 63/110 (57%) who attended follow-up; 14/110 (12.7%) participants had malignancy; no patients upstaged during follow-up. An estimated cost saving of £47,600/10,000 screening CTs performed might be obtained through a conservative approach of short-term interval CT, rather than immediate PET-CT and further investigation.

**Conclusion:**

Early repeat CT avoids PET-CT in more than half of patients with consolidation and can be utilised to reduce over-investigation of screen-detected consolidation, which may demonstrate spontaneous resolution.

**Key Points:**

***Question*** *Is short-term interval follow-up CT in lung cancer screening a safe and cost-effective approach to managing indeterminate (inflammatory or malignant) consolidation?*

***Findings*** *Short-interval CT imaging demonstrates spontaneous resolution of consolidation in over 50% participants in this study, whilst persistent consolidation has a high likelihood of malignancy.*

***Clinical relevance*** *Short-interval CT did not result in upstaging of malignancy and therefore can be considered a safe strategy to prevent the over-investigation of screen-detected consolidation, supporting recent European and American screening recommendations.*

**Graphical Abstract:**

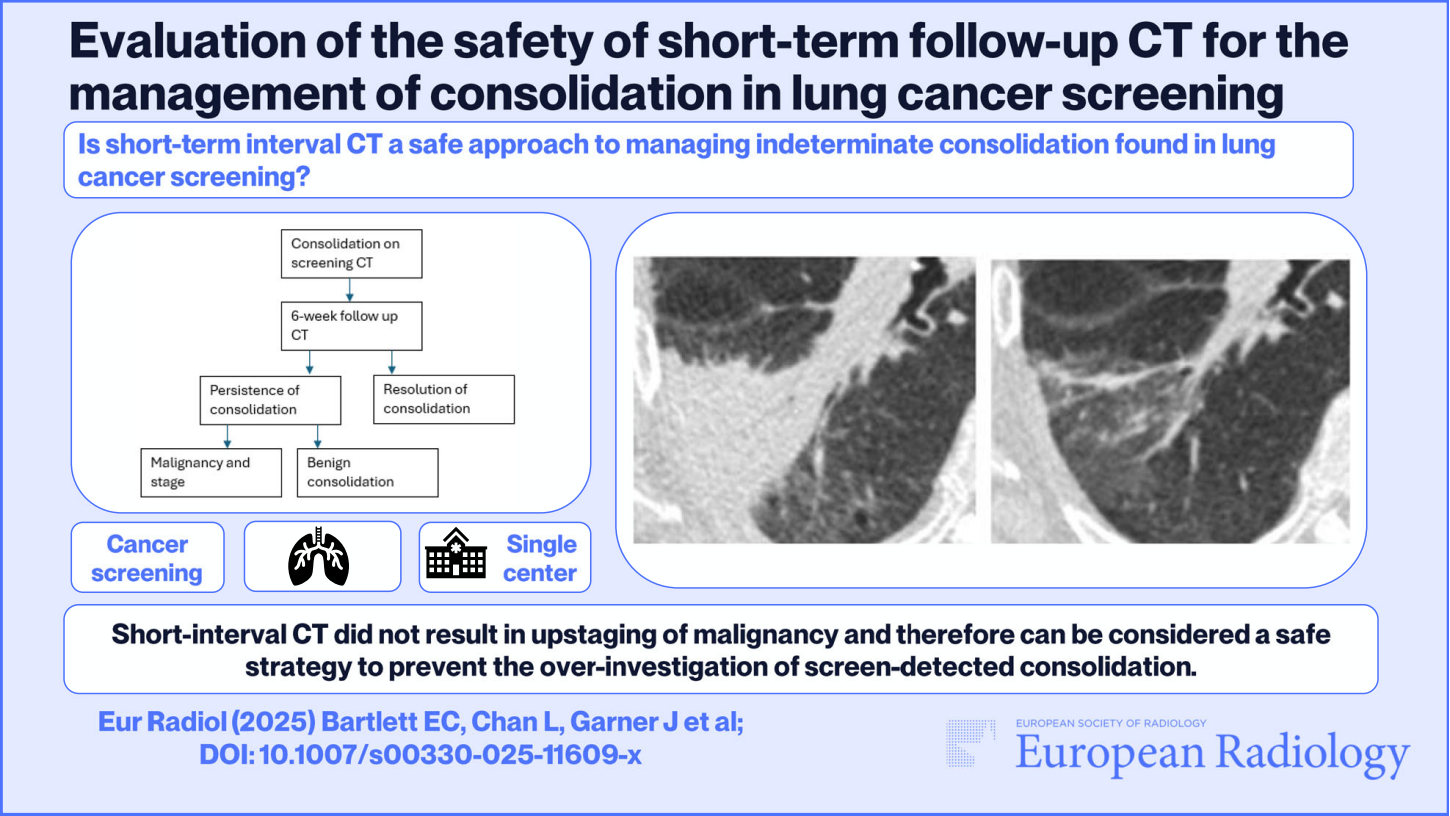

## Introduction

In late 2022, the UK National Screening Committee recommended targeted lung cancer screening (LCS) of high-risk ever-smokers aged 55–74 [1]. In the targeted lung health check (TLHC) programme, nodules detected on low-dose CT (LDCT) are managed using a protocol based on British Thoracic Society (BTS) nodule management guidelines [2, 3]. LDCT occasionally demonstrates consolidation, which may be inflammatory or malignant in aetiology, and appearances on CT may be indeterminate. Current UK screening guidelines suggest multi-disciplinary team (MDT) referral for consolidations suspicious for malignancy but do not make specific ongoing management recommendations [4]. PET-CT imaging in this context is rarely discriminatory; both malignant and infectious consolidation demonstrate Fluorodeoxyglucose (FDG) avidity. Consequently, utilisation of PET-CT may result in sampling of benign disease. Short-term CT imaging may demonstrate resolution of consolidation, avoiding unnecessary PET-CT and invasive procedures. This retrospective study analyses the safety of short-term LDCT follow-up of consolidation in an LCS programme.

## Methods

This retrospective study had institutional review board approval (IRAS 296357). The West London lung cancer screening pilot was a nationally funded service improvement project, the details and baseline results of which have been previously published [5], which was subsequently incorporated into the national TLHC lung screening programme. In brief, ever-smokers, aged 55–74 were invited for a lung health check (LHC), and risk assessment, after which those meeting one or both of two pre-specified risk thresholds (LLPv2 (Liverpool Lung Project v2 (Risk model)) ≥ 2.0% (in the initial pilot), ≥ 2.5% subsequently; PLCOm2012 (Prostate Lung Colorectal and Ovarym2012 (Risk model)) ≥ 1.51%) were offered an LDCT scan. By virtue of being imaged within a screening programme, none of the participants were acutely unwell or symptomatic (with acute cough or fever) at the time of their LDCT scan.

**Fig. 1:**
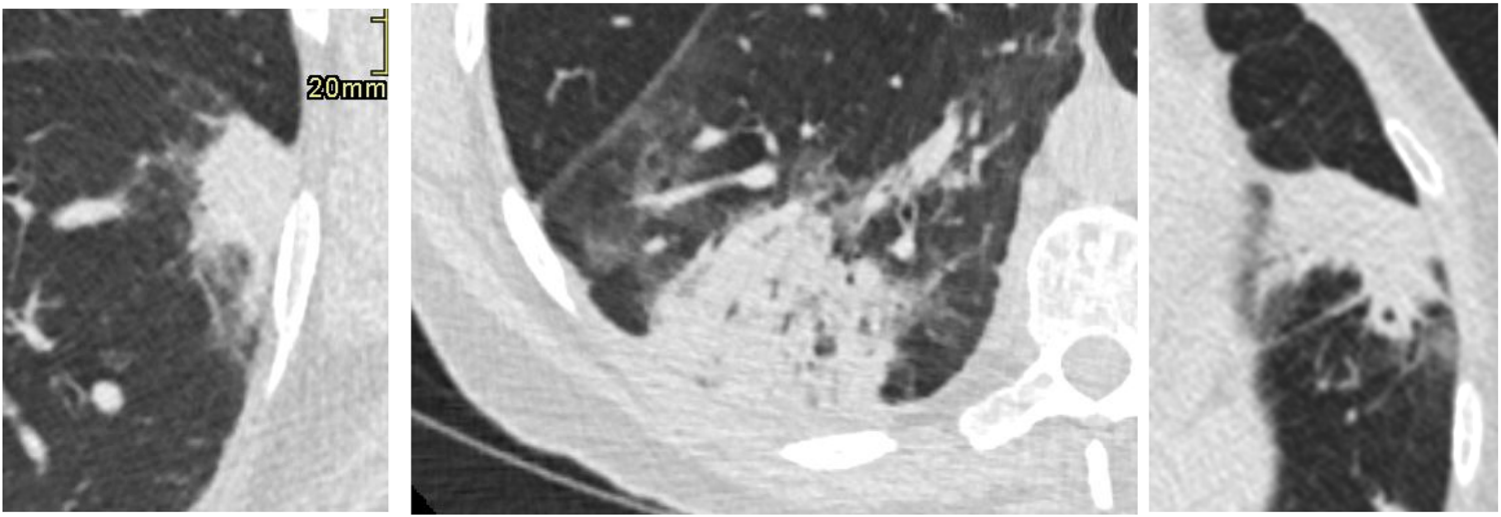
Examples of focal consolidation, for which 6-week follow-up CT was recommended

Participants with indeterminate focal consolidation where there was uncertainty as to whether the aetiology was malignant or inflammatory (Fig. 1) were re-invited for 6-week LDCT without interim treatment. If consolidation was regarded as clearly inflammatory (such as with an associated tree-in-bud pattern), no follow-up CT was undertaken. Participants with mass-like or lobar consolidation highly suspicious of a malignant aetiology were investigated immediately with PET-CT and excluded from this study. Similarly, participants with new consolidation, which developed on a 3-month interval scan, were excluded on the basis that this was highly likely to be inflammatory in aetiology. Persistent consolidation on follow-up CT performed at 6 weeks was investigated with PET-CT or, in some cases, recommended for surveillance at the recommendation of MDT screening radiologists. All LDCT imaging was reported by one of six consultant thoracic radiologists.

## Study outcomes

The study measured (1) the proportion of participants demonstrating consolidation on baseline CT, (2) the proportion of participants with non-resolving consolidation at 6 weeks warranting PET-CT imaging ± subsequent investigation, (3) the percentage of those with consolidation subsequently diagnosed with histologically proven malignancy (4) and the proportion of participants with malignancy deemed to have upstaged during investigation. Upstaging was defined as an opacity categorised as indeterminate, subsequently diagnosed as a Stage II-IV cancer [6] and deemed to have upstaged during surveillance.

Estimates of the costs of an additional LDCT, and the savings from not performing PET-CT, CT biopsy or EBUS at baseline were calculated based on the National Health Service (NHS) National Schedule of NHS costs [7]. Estimated costs of imaging and investigations in this programme were made based on the number of follow-up CT scans, subsequent PET scans and invasive investigations actually performed in the programme. These costs were compared to modelled costs which may have been incurred had all participants with consolidation been investigated with PET imaging at the time of the baseline scan. For modelling purposes, an assumption was made that the same proportion of participants would have had a positive PET study and would have undergone subsequent invasive investigation (CT biopsy or EBUS) as actually happened following the 6-week scan in this study.

## Results

**Fig. 2:**
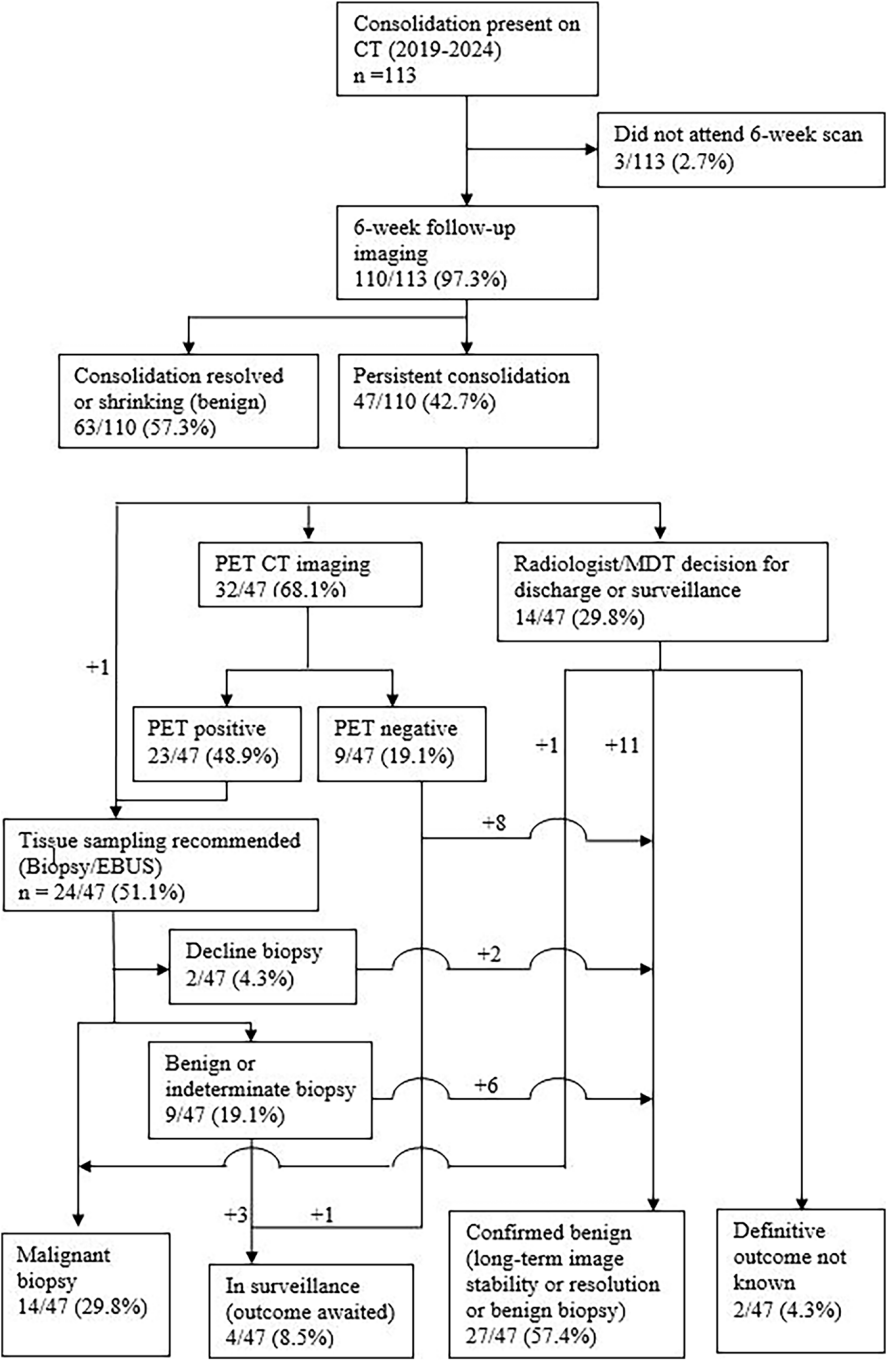
Consort diagram of outcomes of participants with indeterminate focal consolidation

**Fig. 3:**
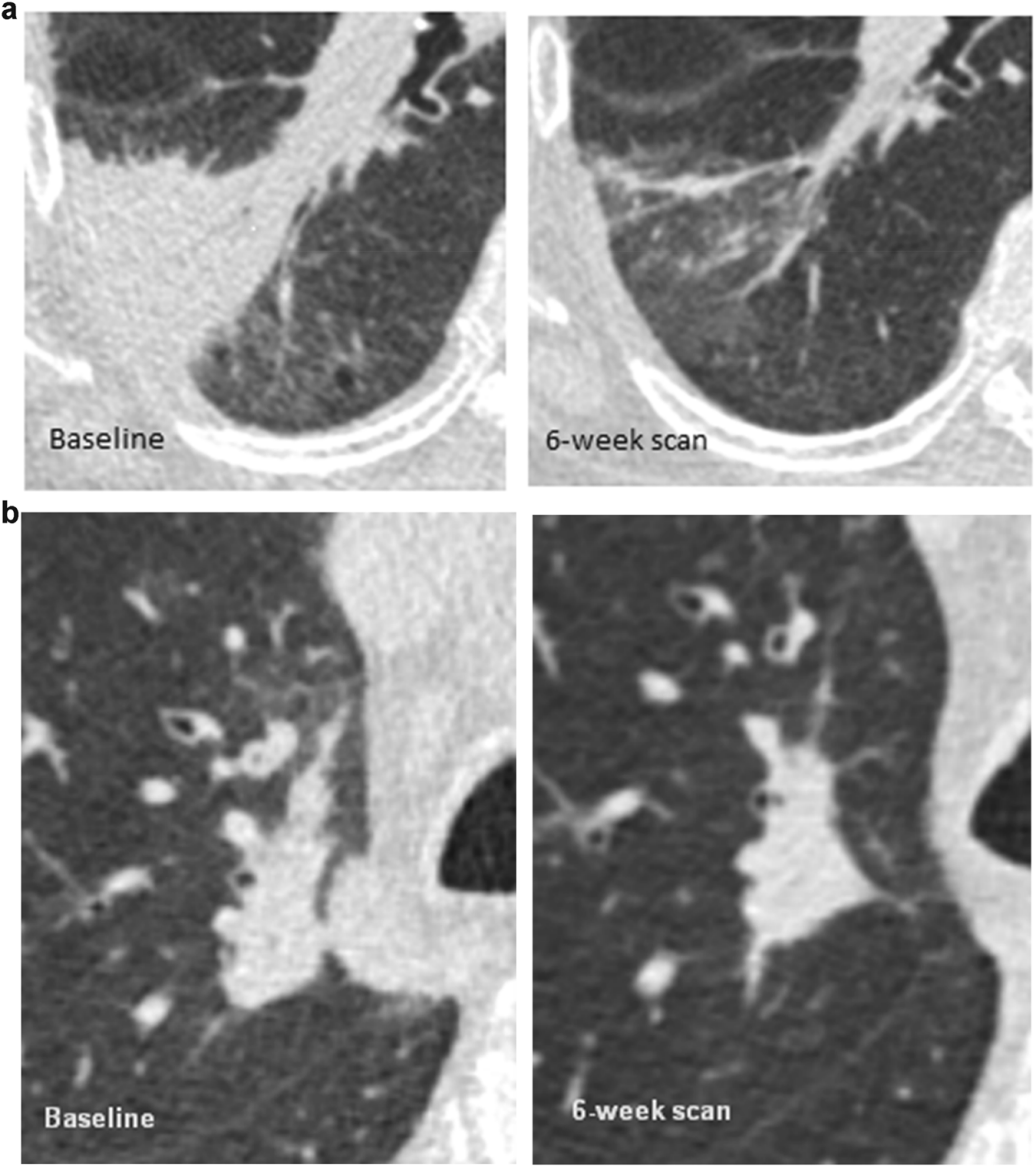
**a** Example of consolidation on a baseline scan (left) showing near complete resolution of at 6-week follow-up imaging (right). **b** Consolidation on a baseline scan (left), with adjacent bronchial thickening on the initial scan suggestive of an inflammatory aetiology but persisting on a 6-week follow-up scan (right); subsequent tissue sampling with bronchoscopic cryobiopsy demonstrated adenocarcinoma

Between January 2019 and January 2024, 10,247 scans were performed in 8778 LCS participants. Consolidation was detected on 113/10,247 (1.1%) scans in 8878 (1.3%) participants (mean age 68 years, range 55–76, 65 males). Most participants (110/113, 97%) attended a 6-week scan. Final outcomes were available in 104/110 (94.5%) participants; long-term outcomes are unknown in 6/110 (5.5%) participants (Fig. 2). Consolidation significantly shrank or completely resolved in 63/110 (57.3%) participants (Fig. 3a). Forty-seven participants had persistent opacities (42.7%) (Fig. 3b). Amongst these, 32/110 (29.1%) underwent PET-CT; one participant (0.9%) had bronchoscopy without a PET-CT. Following PET-CT, invasive investigation was performed in 21/23 (91.3%) participants with a positive PET-CT scan; malignancy was confirmed in 13 cases. Three patients with an indeterminate biopsy result remain under long-term surveillance.

Amongst the 47 participants with persistent consolidation at 6 weeks, consolidation was deemed to be stable by BTS guidelines in 41 cases and growing in 6 cases. In two cases where volumetric measurements were possible, the volume doubling times calculated were 83 days and 153 days. In the remaining 4 cases, the lesion was not accurately segmented by volumetry software but was deemed to be growing by the reporting radiologist. Two of the six growing lesions were subsequently confirmed to be malignant and 4 were benign. Neither of the malignant growing lesions upstaged in the period to follow-up, both Stage IA (Tumour node metastasis (TNM) 8) lesions.

Fourteen participants with persistent consolidation were recommended for further surveillance CT rather than PET-CT based on MDT radiologist evaluation of CT appearances, or for discharge to the next screening round based on MDT consensus. Stability over more than 1 year of follow-up confirmed benignity in 10/14. Three participants have not had further long-term imaging. One participant was discharged as the CT morphology was suggestive of a scar and later presented with a  carcinoma within a scar (Stage IB).

Amongst those for whom final outcomes are available, the risk of malignancy in those with indeterminate consolidation on the initial scan was 14/104 (13.5%), rising to 14/47 (29.8%) in those with non-resolving consolidation (Stage IA, *n* = 8; Stage IB, *n* = 2; Stage IIA, *n* = 3; Stage 1E, *n* = 1 (lymphoma)). None of the Stage IIA malignancies (Tumour node metastasis (TNM) 8) were deemed to have upstaged over the 6-week interval to repeat CT.

Table 1 demonstrates (1) the estimated actual costs of interval follow-up CT and the associated investigations performed in this programme and (2) models the potential cost of investigations had all patients undergone PET-CT following baseline imaging. Of those who had PET-CT imaging at 6 weeks, invasive investigations were recommended in 23/32 (71.9%). CT biopsy was recommended in 20/32 (62.5%) participants, and EBUS in 3/32 (9.4%) participants; modelling is based on these proportions.

**Table 1:** Estimated actual costs associated with PET-CT and subsequent invasive investigations in this study, based on NHS reference costs (2023/2024) [7] as well as estimated potential costs of initial and subsequent investigations had all patients undergone PET-CT following identification of consolidation at baseline imaging

Activity or investigation	Cost/unit (£)	Number of participants	Total cost (£)
Estimated actual costs of investigations for 113 participants reported in this study			
Interval CT follow-up	109.00	110	11,990.00
PET-CT^a^	561.00	32	17,952.00
Percutaneous CT biopsy	187.00	18^b^	3366.00
EBUS	899.00	3	2697.00
Total			36,005.00
Estimated modelled potential costs associated with investigation with PET and biopsy following initial CT imaging			
PET-CT^a^	561.00	110	61,710.00
Percutaneous CT biopsy	187.00	69	12,903.00
EBUS	899.00	10	8990
Total			83,603.00

^a^ Costs are based on PET-CT of 2 or 3 areas of the body

^b^ Two participants declined CT biopsy

This estimate suggests that, for every 10,000 CTs performed in screening, a short-term follow-up CT protocol for indeterminate consolidation could save approximately £47,600 (€57,480.00) by avoiding PET-CT and subsequent invasive procedures.

## Discussion

Consolidation is an infrequent finding in asymptomatic LCS populations (1.3% in this study), with over 50% resolving or shrinking at 6 weeks without treatment, indicative of a benign aetiology. However, in this study, just under one-third of those with persistent consolidation after 6 weeks had malignancy. Persistent consolidation therefore warrants further investigation with PET-CT or MDT review in the first instance. In this study, this approach of short-interval CT follow-up did not lead to an interim stage shift.

In 2022, the Lung Imaging Reporting and Data System (Lung-RADS) used for the classification of lung screening findings in the United States, added “findings suggesting an indeterminate infective of inflammatory process”, to management guidelines with a recommendation for a follow-up CT at 1–3 months (Lung-RADS 0) [8, 9]. A recent European clinical practice statement also recommends short-interval follow-up CT for indeterminate consolidation [10]. Our study confirms that a short-term follow-up CT after identification of consolidation appears to be a reasonable method of distinguishing persistent potentially malignant versus resolving inflammatory consolidation.

The Early Lung Cancer Action Project found that short-interval CT with interim antibiotic treatment resulted in a similar resolution rate (51.3%) of consolidation and a slightly higher malignancy rate (43.6%) among those with non-resolving consolidation [11]. Another study of LCS participants reported a substantially lower malignancy rate (0.6%), likely due to the inclusion of more obviously inflammatory cases [12].

PET-CT imaging is of higher cost than unenhanced CT imaging [7], and subsequent invasive investigation for benign lesions is not only costly but can also cause significant anxiety to participants. PET-CT is also associated with a substantial radiation dose, in the region of 10–15 mSv for a whole-body PET-CT [13], compared to 1–1.15 mSv for a low-dose thoracic CT [14]. The benefits of an approach using short-interval CT for follow-up are therefore a reduction in unnecessary further investigations and costs and a reduction in radiation dose to participants. The costs of diagnosis and investigation go beyond those calculated here, including physician and MDT time. The study may underestimate the costs of PET-CT imaging and further investigation due to the possibility that more opacities would have been FDG avid at baseline due to inflammation. Thus, the potential savings are likely underestimated.

In this study, the evaluation of indeterminate focal consolidation warranting a 6-week CT was based on the judgement of the reporting radiologist. Therefore, the applicability of these results may vary according to reader expertise and judgement. The results do not apply to consolidation identified at 3-month nodule follow-up CT, which was not evaluated in this study, nor to clearly mass-like or lobar consolidation, which should be investigated more promptly. In our study, we required long-term stability over more than 1 year, or a MDT confirmed true negative diagnostic biopsy to be confident of benignity. Consequently, a limitation of this study is that long-term follow-up data are lacking for 5.5% of participants who attended a 6-week follow-up.

In conclusion, short-interval LDCT imaging for focal indeterminate consolidation in LCS is a cost-effective and safe strategy that reduces unnecessary investigation of inflammatory spontaneously resolving abnormalities. This could be a reasonable approach in a national screening programme, thereby reducing costs of investigation whilst ensuring appropriate follow-up of indeterminate consolidation.

## Abbreviations

BTS: British Thoracic Society
CT: Computed tomography
EBUS: Endobronchial ultrasound
FDG: Fluorodeoxyglucose
LCS: Lung cancer screening
LDCT: Low-dose CT
Lung-RADS: Lung Imaging Reporting and Data System
MDT: Multi-disciplinary team
NHS: National Health Service
PET-CT: Positive emission tomography–computed tomography
TLHC: Targeted lung health check
TNM: Tumour node metastasis (Lung cancer staging)
UK: United Kingdom
